# Left ventricular electrical potential measured by the NOGA XP electromechanical mapping method as a predictor of response to cardiac resynchronization therapy

**DOI:** 10.3389/fcvm.2023.1107415

**Published:** 2023-05-04

**Authors:** Jacek Wilczek, Tomasz Jadczyk, Wojciech Wojakowski, Krzysztof S. Gołba

**Affiliations:** ^1^ Department of Electrocardiology and Heart Failure, Medical University of Silesia, Katowice, Poland; ^2^ Electrocardiology Department, Upper Silesian Medical Center, Katowice, Poland; ^3^ Division of Cardiology and Structural Heart Diseases, Medical University of Silesia, Katowice, Poland; ^4^ Interventional Cardiac Electrophysiology Group, International Clinical Research Center, St. Anne's University Hospital in Brno, Brno, Czech Republic; ^5^ Third Department of Cardiology, Upper Silesian Medical Center, Katowice, Poland

**Keywords:** cardiac pacing, resynchronization therapy, cardiac mapping, cardiac electrical potential, heart failure

## Abstract

**Objectives:**

The aim of the study was to determine whether left ventricular electrical potential measured by electromechanical mapping with the NOGA XP system has predictive value for response to CRT.

**Background:**

Approximately 30% of patients who undergo cardiac resynchronization therapy do not see the expected effects.

**Methods:**

The group of 38 patients qualified for CRT implantation were included in the study, of which 33 patients were analyzed. A 15% reduction in ESV after 6 months of pacing was used as a criterion for a positive response to CRT. The mean value and sum of unipolar and bipolar potentials obtained by mapping with the NOGA XP system and their predictive value in relation to the effect of CRT were analyzed using a bulls-eye projection at three levels: 1) the global value of the left ventricular (LV) potentials, 2) the potentials of the individual LV walls and 3) the mean value of the potentials of the individual segments (basal and middle) of the individual LV walls.

**Results:**

24 patients met the criterion of a positive response to CRT vs. 9 non-responders. At the global analysis stage, the independent predictors of favorable response to CRT were the sum of the unipolar potential and bipolar mean potential. In the analysis of individual left ventricular walls, the mean bipolar potential of the anterior and posterior wall and in the unipolar system, mean septal potential was found to be an independent predictor of favorable response to CRT. In the detailed segmental analysis, the independent predictors were the bipolar potential of the mid-posterior wall segment and the basal anterior wall segment.

**Conclusions:**

Measurement of bipolar and unipolar electrical potentials with the NOGA XP system is a valuable method for predicting a favorable response to CRT.

## 1. Introduction

Heart failure has one of the highest morbidity and mortality rates in the world. It is estimated that about 2% of global population is affected by symptomatic heart failure, and this percentage increases with age. Symptoms of the disease appear in 10% of people over 70. The 12-month total mortality rate for heart failure among inpatients and stable/outpatients is 17% and 7%, respectively, and the 12-month incidence of hospitalization is 44% and 32%, respectively. The pathomechanism of heart failure is complex. ECG changes are observed in some patients with significantly reduced ejection fraction (EF < 35%) in the form of atrioventricular conduction abnormalities and widened QRS complex, about 35% of whom manifest left bundle branch block. Conduction disturbances underlie cardiac systolic dyssynchrony and cause hemodynamic consequences that lead to adverse remodeling. Cardiac resynchronization therapy (CRT) restores the normal heart contraction sequence, improves the depolarization pattern, has a beneficial effect on quality of life, reduces the severity of symptoms, morbidity and mortality rates. Cardiac function is based on a complex electro-mechanical coupling mechanism. Systolic function deterioration is not only a result of the impaired propagation of the electrical impulse in the heart, but also of the loss of active cardiomyocytes capable of automatic resting depolarization and generating electrical potential. This raises the question of whether the value of this potential can predict the effect of resynchronization. The present study aimed to determine whether left ventricular (LV) electrical potential, as examined by electromechanical mapping with the NOGA XP system, has predictive value in responding to resynchronization therapy.

## 2. Methods

Between April 2014 and July 2017, the study enrolled 38 patients, including 9 women (24%) and 29 men (76%), aged 49 to 76 years (mean age of 65.6 ± SD 5.7), with sinus rhythm, left bundle branch block and ischemic cardiomyopathy, with ESC class I and II indications for resynchronization system implantation (CRTD). Exclusion criteria were defined as follows: acute coronary syndrome for less than 3 months prior to the study inclusion, coronary artery disease requiring revascularization, previously implanted pacemaker or cardioverter-defibrillator, aortic valve calcification or LV thrombus, chronic kidney disease, pregnancy or lactation, active malignancy, viral infection, bleeding diathesis, allergy to contrast agent, current participation in another study, life expectancy less than 6 months. The project was prospective, single cohort study compliant with the Declaration of Helsinki. It was approved by the Bioethics Committee of the Silesian Medical University in Katowice KNW/0022/KB1/17/15. All patients signed an informed consent form. The characteristics of the patient group is shown in Table 1.

**Table 1:** Characteristics of the study group.

Age		65.75 ± 5.95
Gender: Female/Male		7 (21%)/26 (79%)
Weight		86.36 ± 15
BMI		30.23 ± 4.3
Etiology HF (ischemic)		33 (100%)
Medications	B-blocker	33 (100%)
	ACEI/ARB	33 (100%)
	Spirinolactone/eplerenone	31/33 (94%)
	Loop diuretic	27/33 (82%)
	Statine	32/33 (97%)
Comorbidities	Previous myocardial infarction	30 (91%)
	Hypertension	31 (94%)
	Diabetes mellitus	16 (48%)
	Chronic Obstructive Pulmonary Disease	2 (6%)
	Atrial fibrillation	7 (2.94%)
% BiV pace at 6MFU	97 ± 3.7 (%)	

ACEI, angiotensin-converting enzyme inhibitors; ARB, angiotensin receptor II blockers; %BiV pace, % biventricular pacing; BMI, body mass index; 6MFU, six month follow-up.

**Figure 1:**
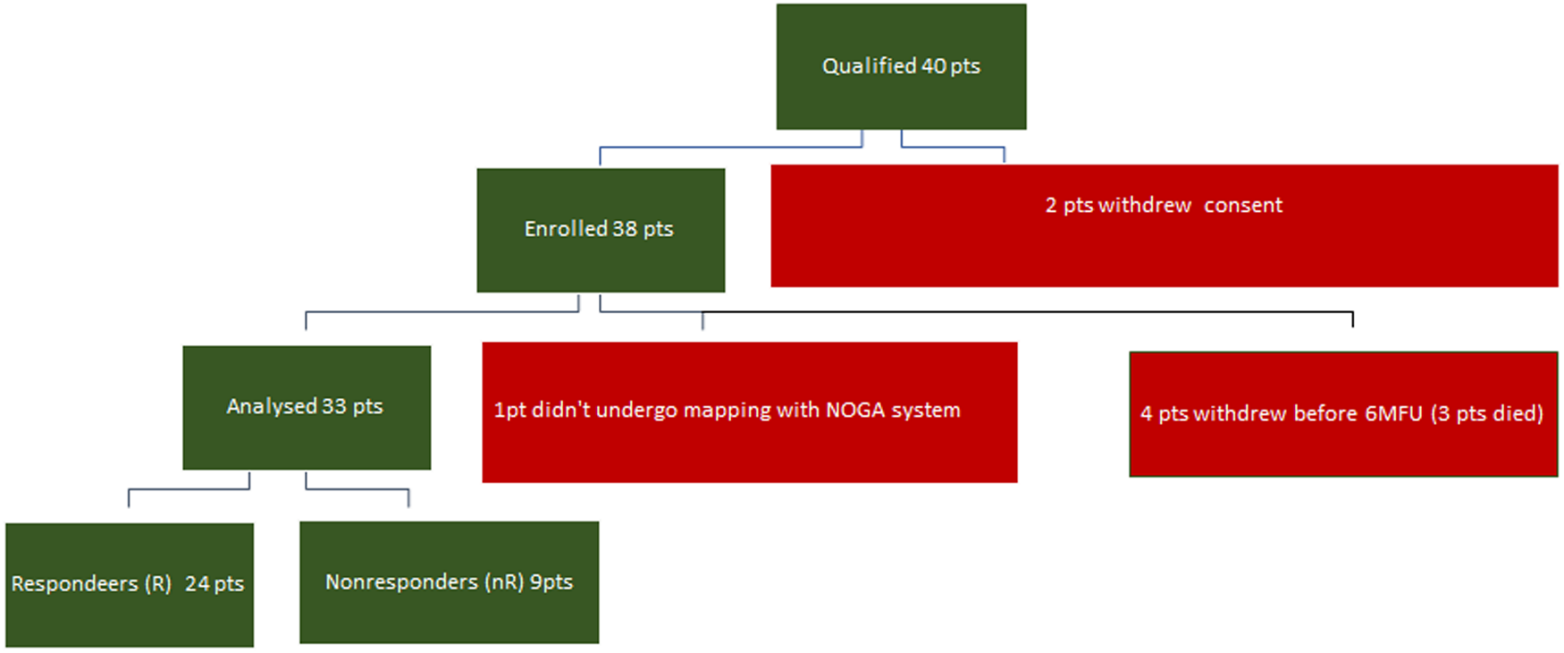
Study group.

Thirty-three patients were analyzed; one patient could not be mapped with the NOGA system, and four withdrew before the 6-month follow-up examination (6MFU) (Figure 1).

All patients qualified for implantation of the resynchronization system underwent echocardiography using the Phillips iE 33 and Cx 50 devices. End-systolic volume (ESV), left ventricular ejection fraction (LVEF), end-diastolic volume (EDV), and the degree of mitral regurgitation were assessed. A minimum 15% reduction in ESV after 6 months of follow-up was used as a criterion for evaluating the response to resynchronization therapy as positive. A clinical evaluation was also conducted, including the NYHA cardiovascular fitness classification, 6MWT. Electromechanical mapping was performed once in each patient using the NOGA XP AEMM system (anatomo-electromechanical mapping system NOGA XP®, Biologic Delivery Systems, Division of Biosense Webster, a Johnson & Johnson Company, Irwindale, California). Left ventricular electrical potentials were recorded in unipolar and bipolar systems (273 ± 47 recorded and mapped points per patient). The analysis of the averaged potentials and their sum was performed in the LV bull-eye projection divided by basal segments (anterior, septal, posterior and lateral), analogous intermediate segments (anterior, septal, posterior and lateral) and in one apical segment. The analysis was performed for the entire left ventricle, individual walls (anterior, lateral, posterior and septal) and individual basal and intermediate segments. The resynchronization system implantation procedure was performed in all patients. The right ventricular lead was located in the RV apex. Implantation of the left ventricular lead in all patients was performed in a standardised standard manner, with the selection of the target lateral or posterolateral vein avoiding the location in the apical region (1). The result of electromechanical mapping did not determine the location of the electrode. This allowed the assessment of the value of electrical potential as a predictor of the CRT response at the standard left ventricular lead location on the basis of research studies and consequently from guidelines (1, 2). The function of the resynchronization system was confirmed four weeks after the implantation procedure. Echocardiography and clinical evaluation were performed again 6 months after the implantation.

## 3. Statistical analysis

Statistical analysis involved the Shapiro-Wilk test in assessing normality of distribution, Student's *t*-test for independent samples, and Whitney's *U*-Mann to compare groups. A weighted unidimensional linear regression analysis was used to assess the association between selected parameters. A model based on logistic regression analysis, C-statistics, and ROC analysis was used for predictive evaluation. Calculations were performed using MedCalc® statistical software, version 2.114–32-bit.

## 4. Results

The criterion for a positive response to resynchronization therapy was met by 24 analyzed patients (73%). Changes in echocardiographic and clinical parameters after 6 months concerning baseline values are shown in Table 2.

**Table 2:** Change in echocardiographic and clinical parameters after 6 months of resynchronization pacing.

Parameter		Base	6MFU	*P* value
LVESV		182.03 ± 42.22	132.6 ± 56.84	*P* < 0.001
LVESVi		92.47 ± 21.09	71.88 ± 29.73	*P* < 0.001
LVEF		28.11 ± 4.57	38.32 ± 9.84	*P* < 0.001
LVEDV		254 ± 52.39	226.36 ± 73.67	*P* = 0.006
LVEDVi		129.08 ± 25.89	114.36 ± 38.06	*P* = 0.003
MR moderate		16 (48.5%)	2 (6.06%)	Number of positive differences 13 Number of negative differences 0 Number of no change 20
NYHA class	I	0 (0.00%)	4 (12.12%)	*p* < 0.001 Number of positive differences 26 Number of negative differences 0 Number of no change 7
	II	9 (27.3%)	7 (81.82%)	
	III	24 (72.7%)	2 (6.06%)	
6MWT [m]		354 ± 111	422 ± 103	*P* < 0.001

LVEDV, left ventricular end-diastolic volume; LVEDVi, left ventricular end diastolic volume index; LVEF, Left ventricle ejection; LVESV, left ventricular end-systolic volume; LVESVi, left ventricular end systolic volume index; MR, mitral regurgitation; 6MWT, 6 minute walk test.

**Figure 2:**
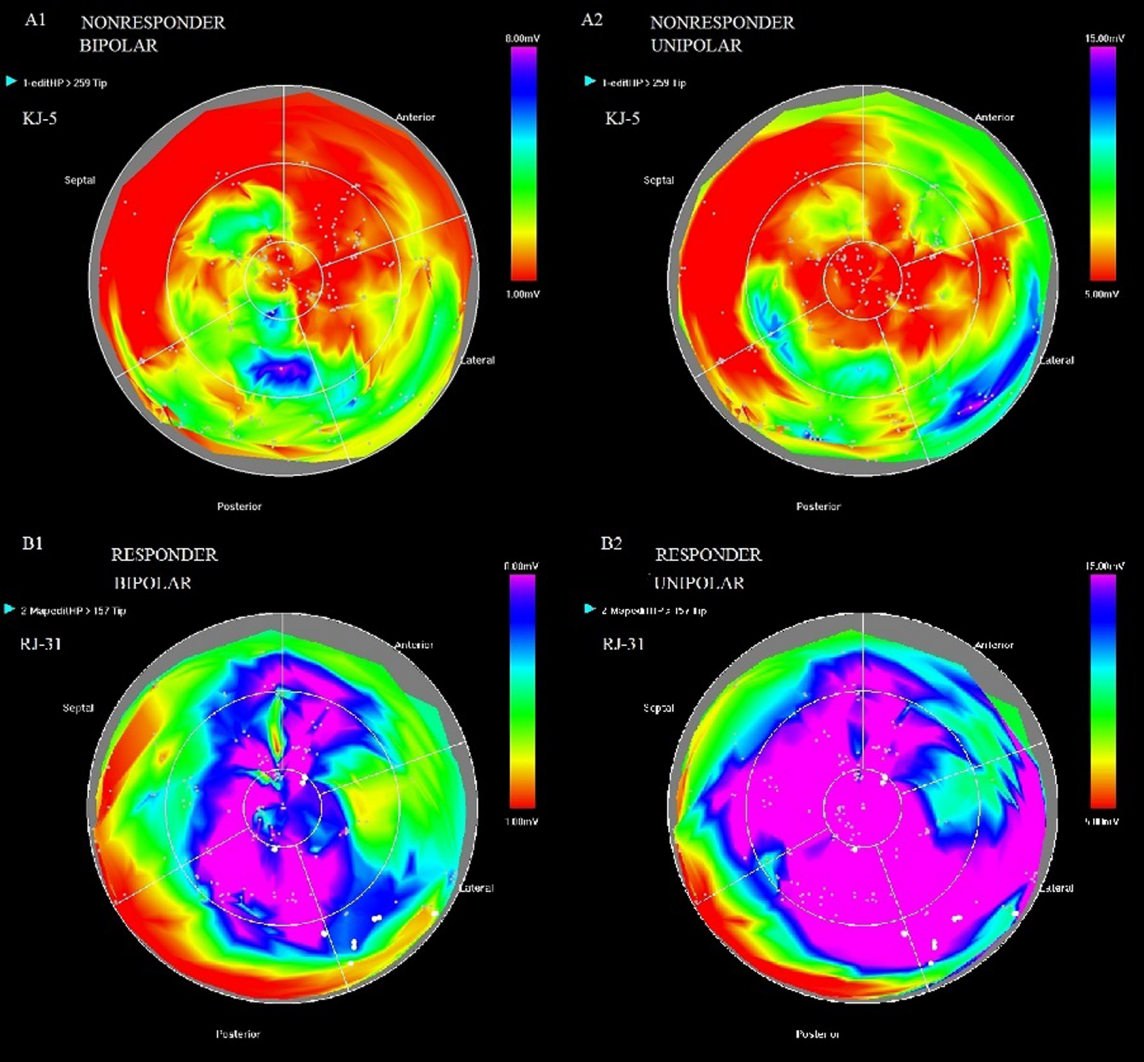
Bipolar (1) and unipolar (2) potential maps in patient KJ-5-non-responder (**A**) and patient RJ-31-responder (**B**).

An example of bipolar (1) and unipolar (2) potential maps in a non-responder (A) and a responder (B) is shown in Figure 2.

## 5. General analysis of potentials in unipolar and bipolar configurations

The values of mean potentials and the sum of mean potentials measured in 9 segments in the responder and non-responder groups were compared, obtaining a significant difference between compared groups except for the mean value of the unipolar potential. The values of LV electrical potential in the responders and non-responders groups are shown in Table 3.

**Table 3:** Lv electric potential values in responders and non-responders group.

	Potential			
	Bipolar mean	Bipolar sum	Unipolar mean	Unipolar sum
All (*n* = 33)	2.78 ± 1.23	24.92 ± 10.83	10.03 ± 5.89	86.5 ± 23.58
Responder A	3.3 ± 1.2	29.61 ± 10.72	10.86 ± 2.55	97.02 ± 21.91
Nonresponder B	1.98 ± 0.69	17.69 ± 6.15	8.75 ± 3.5	70.31 ± 15.95
Test-*t p* value	0.0014	0.0010	0.209	0.0007

Regression analysis showed a clear correlation between the mean values of the potentials measured in the 9 segments in the bipolar configuration (*r* = 0.45, *p* = 0.009), as well as their sum (*r* = 0.41, *p* = 0.017) and the percentage of change in the end-systolic volume after 6 months of resynchronization pacing relative to baseline. Such a relationship was also found for the mean values of potentials (*r* = 0.35, *p* = 0.05) and the sum of potentials measured in the 9 segments in the unipolar configuration (*r* = 0.39, *p* = 0.023). Logistic regression analysis and ROC analysis found clear predictive value of mean unipolar (sensitivity 90%, specificity 69.2%, cut-off value > 8.2 mV) and bipolar (sensitivity 85%, specificity 76.9%, cut-off value > 2.15 mV) and summed unipolar (sensitivity 90%, specificity 76.9%, cut-off value > 73.7 mV) and bipolar (sensitivity 85%, specificity 76.9%, cut-off value > 18.94 mV) LV electrical potentials of positive response to resynchronization therapy.

Based on the results, the prediction models were developed for the individual parameters of LV electrical potentials in unipolar and bipolar configurations. The analysis revealed that the sum of recorded unipolar potentials—unipolar sum (AUC 0.8465, 95% CI 0.67–0.95, *p* = 0.001), as well as bipolar mean potential (AUC 0.835, 95%, CI 0.67–0.94, *p* = 0.001) could be an independent predictor of a positive response to the resynchronization therapy.

## 6. Analysis of potentials in unipolar and bipolar configurations of the individual walls of the left ventricle in the long axis

**Figure 3:**
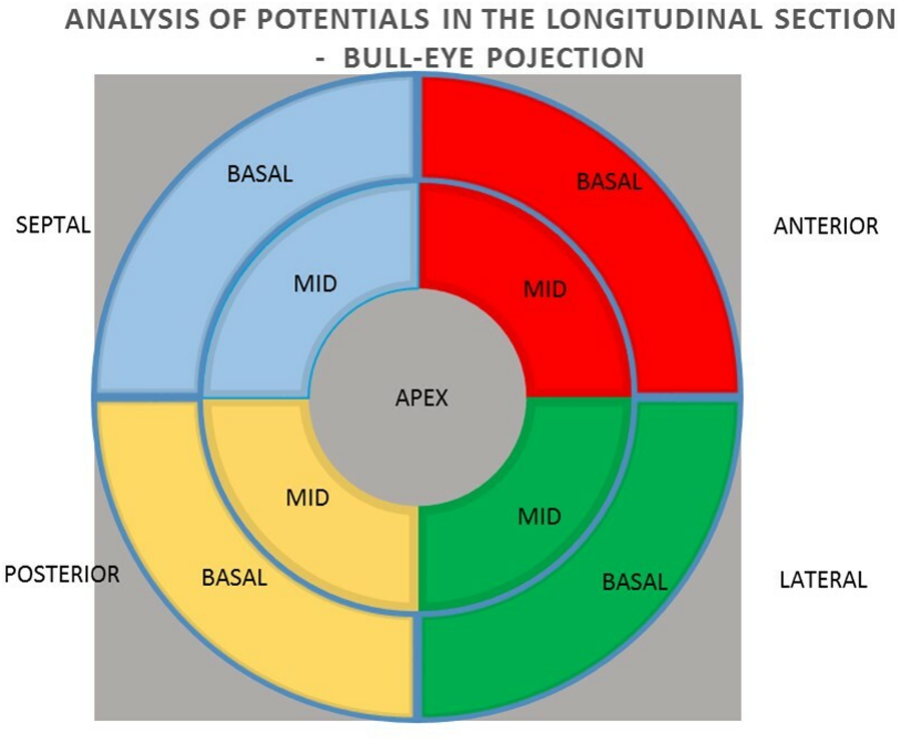
Arrangement of individual walls in longitudinal section in bull-eye projection.

In the next step, the relationships between the potential values in the unipolar and bipolar configurations of the segments in the longitudinal section of anterior, lateral, posterior, and septal walls were analyzed for the summed mean values of the basal and middle segments (Figure 3).

In analyzing individual wall potentials in the long axis, differences were observed between responder and non-responder group in sum and mean unipolar potentials, as well as bipolar potentials of the anterior, posterior and septal walls. Regarding the lateral wall, the difference occurred in unipolar potentials and tended toward significance regarding the bipolar configuration, as in Table 4.

**Table 4:** Differences in mean and summed values of the anterior, lateral, and posterior walls and septal potentials in unipolar and bipolar configurations in responder (R) and non-responder (nR) group.

	BIPOLAR		UNIPOLAR	
Status	R	nR	R	nR
Suma Anterior	5.22 ± 3.32	3.14 ± 1.66	20.76 ± 5.73	15.39 ± 5.64
*P* value	0.045		0.013	
Mean Anterior	2.61 ± 1.66	1.57 ± 0.83	10.38 ± 2.86	7.69 ± 2.82
*P* value	*P* = 0.045		0.013	
Suma Lateral	7.55 ± 3.9416	5.12 ± 3.0608	24.67 ± 7.45	17.06 ± 6.31
*P* value	0.069		0.005	
Mean Lateral	3.77 ± 1.97	2.56 ± 1.53	12.33 ± 3.72	8.5322 ± 3.16
*P* value	0.069		0.005	
Suma Posterior	10.04 ± 4.83	4.90 ± 3.09	25.06 ± 8.31	16.40 ± 7.42
*P* value	0.002		0.005	
Mean Posterior	5.02 ± 2.42	2.45 ± 1.54	12.53 ± 4.15	8.20 ± 3.71
*P* value	0.002		0.005	
Suma Septal	4.54 ± 2.78	2.42 ± 1.38	17.39 ± 5.49	11.00 ± 4.47
*P* value	0.016		0.001	
Mean Septal	2.27 ± 1.39	1.21 ± 0.69	8.70 ± 2.75	5.50 ± 2.23
*P* value	0.016		0.001	

In logistic regression analysis confirmed by C-statistic analysis, the predictive factors of positive response to resynchronization therapy include mean bipolar potentials of the posterior wall (AUC 0.808, 95% CI 0.633–0.923, *p* = 0.008) and the septum (AUC 0.738, 95% CI 0.504 to 0.838, *p* = 0.027). These parameters tended to be significant in the anterior (AUC 0.688, 95% CI 0.633–0.923, *p* = 0.067) and lateral (AUC 0.70, 95% CI 0.516–0.846, *p* = 0.080) walls. The mean potential in the unipolar configuration of the anterior wall (AUC 0.746, 95% CI 0.565–0.881, *p* = 0.003), lateral wall (AUC 0.781, 95% CI 0.603–0.905, *p* = 0.010), posterior wall (AUC 0.792, 95% CI 0.616–0.913, *p* = 0.010) and septal wall (AUC 0.812, 95% CI 0.638–0.926, *p* = 0.010) also show predictive value. A predictive model was developed for the mean potential values of each left ventricular wall in bipolar and unipolar configurations. Mean bipolar anterior and posterior wall potential, as well as mean septal potential in unipolar configuration, appeared to be independent predictors of favorable response to the resynchronization therapy, noted in Table 5.

**Table 5:** Predictive model based on mean wall potentials in bipolar and unipolar configurations.

BIPOLAR			
ROC (AUC) 0.881 95% CI 0.720–0.967 *P*<<0.0001			
VARIABLE	ODDS RATIO	95% CI	*p*
MEAN BIPOLAR ANTERIOR	**3**.**9402**	**1.17–13.22**	**0**.**03**
MEAN BIPOLAR POSTERIOR	**2**.**0659**	**1.09–3.91**	**0**.**03**
MEAN BIPOLAR LATERAL	0.8851	0.49**–**1.60	0.69
MEAN BIPOLAR SEPTAL	1.4457	0.57**–**7.69	0.27
UNIPOLAR			
ROC (AUC) 0.869 95% CI 0.706–0.961 *P*<<0.0001			
VARIABLE	ODDS RATIO	95% CI	*p*
MEAN UNIPOLAR ANTERIOR	1.2198	0.89**–**2.48	0.13
MEAN UNIPOLAR POSTERIOR	1.0600	0.80**–**1.57	0.50
MEAN UNIPOLAR LATERAL	1.0310	0.71**–**1.59	0.77
MEAN UNIPOLAR SEPTAL	**1**.**8005**	**1.14–2.83**	**0**.**01**

The ROC analysis confirms that the predictors of a positive response to CRT include mean anterior wall potential (criterion > 1.93, sensitivity 55.0, specificity 76.9) and mean posterior wall potential (criterion > 3.55, sensitivity 75.0, specificity 84.6) while for the unipolar system, the mean septal potential (criterion > 6.56, sensitivity 75.0, specificity 76.9).

## 7. Analysis of potentials in unipolar and bipolar configurations of individual segments in the left ventricular cross-section

**Figure 4:**
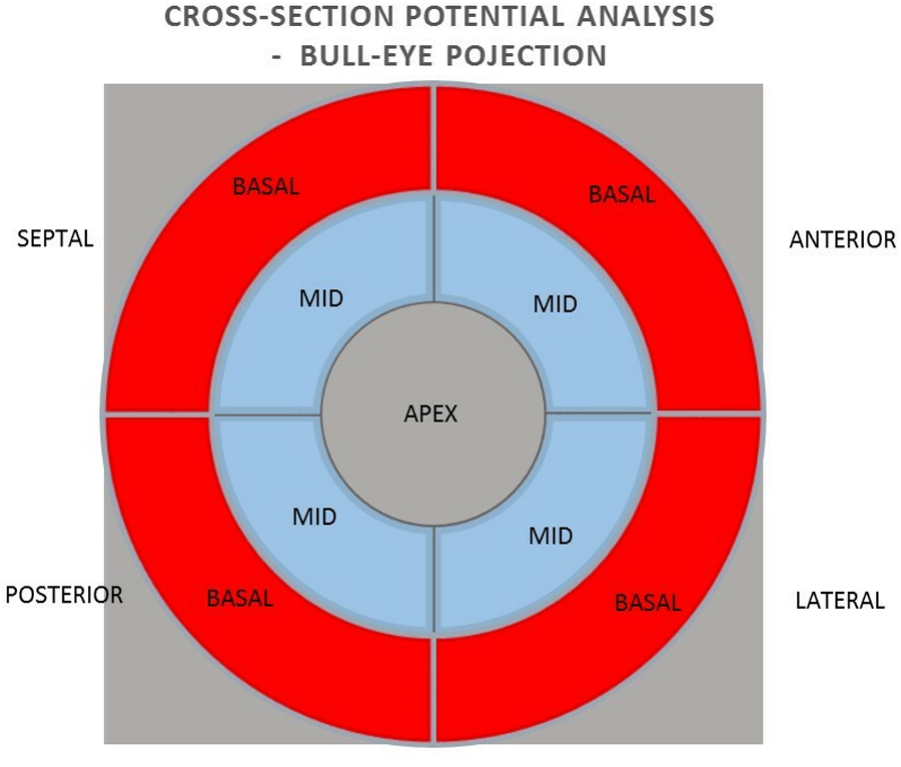
Arrangement of heart wall segments in cross-section in bull-eye projection.

In the next step, the relationships between the values of potentials in unipolar and bipolar configurations of individual basal and medial segments in cross sections of the anterior, lateral, posterior and septal walls were analyzed (Figure 4).

Differences between the values of potentials in responders (R) and non-responders (nR) in individual transverse segments in the study group were observed only during measurements in the bipolar configuration. The exceptions were the middle segment of the anterior wall and the middle segment of the lateral wall, in which no difference was observed (Table 6).

**Table 6:** Potential values of LV segments in unipolar and bipolar configurations in responder (R) and non-responder (nR) groups.

	Mid BI		Basal BI		Mid UNI		Basal UNI	
Status	R	nR	R	nR	R	nR	R	nR
Mean all	3.37 ± 1.27	2.01 ± 9.65	3.46 ± 1.56	1.88 ± 1.01	10.44 ± 3.47	8.96 ± 2.82	9.69 ± 3.25	8.94 ± 2.64
*P* value	0.001		0.003		0.250		0.540	
Anterior	2.018 ± 1.88	1.64 ± 1.14	3.04 ± 1.95	1.49 ± 1.22	9.48 ± 4.56	8.52 ± 3.29	9.58 ± 3.64	9.11 ± 2.73
*P* value	0.370		0.016		0.560		0.710	
Lateral	3.24 ± 2.14	2.57 ± 1.77	4.31 ± 2.43	2.55 ± 1.75	10.69 ± 4.03	9.74 ± 3.58	11.68 ± 4.8	10.93 ± 3.89
*P* value	0.360		0.030		0.530		0.670	
Posterior	5.47 ± 2.76	2.45 ± 1.53	4.58 ± 3.04	2.45 ± 1.62	12.76 ± 4.72	10.87 ± 5.46	10.37 ± 3.91	9.18 ± 3.28
*P* value	0.001		0.028		0.330		0.410	
Septal	2.63 ± 1.66	1.39 ± 0.97	1.91 ± 1.44	1.02 ± 0.79	8.83 ± 3.55	6.72 ± 2.90	7.11 ± 3.71	6.54 ± 2.06
*P* value	0.002		0.047		0.120		0.660	

In the regression analysis confirmed by the results of the C-statistic, the predictive factors of a positive response to the resynchronization therapy in the analysis of transverse segment potentials include the mean value of the bipolar potential of all basal segments (AUC 0.808, 95% CI 0.633 to 0.923, *p* = 0.010), as well as the mean value of the bipolar potential of all middle segments (AUC 0.835, 95% CI 0.665 to 0.941, *p* = 0.008). In the analysis of individual segments, the bipolar potentials of the mid-segment of the posterior wall (AUC 0.819, 95% CI 0.647 to 0.931, *p* = 0.009) and the basal segment of this wall show a predictive relationship with respect to a favorable response to CRT (AUC 0.712, 95% CI 0.528 to 0.855, *p* = 0.040), as well as the basal segment of the anterior wall (AUC 0.796, 95% CI 0.557 to 0.875, *p* = 0.040) and lateral wall (AUC 0.738, 95% CI 0.557 to 0.855, *p* = 0.040). The bipolar signal of the medial septal segment also shows predictive value in this regard (AUC 0.735, 95% CI 0.553 to 0.872, *p* = 0.035), while the bipolar signal of the inferior part of the septum tends to be significant in this regard (AUC 0.708, 95% CI 0.524 to 0.852, *p* = 0.07). No predictive value was found in response to resynchronization therapy for the anterior and lateral medial segments.

A predictive model of the response to the resynchronization therapy based on the bipolar potential of individual middle and basal segments was developed. It was found that the independent predictors of a positive response to the resynchronization therapy included the bipolar potential of the basal segment of the anterior wall and the bipolar potential of the middle segment of the posterior wall (Table 7).

**Table 7:** Predictive model for a positive response to the resynchronization therapy based on the bipolar potential of individual middle and basal segments.

ROC (AUC) 0.888 95% CI 0.730–0.971 *p* < 0.0001			
VARIABLE	ODDS RATIO	95% CI	*p*
ANTEROBASAL BIPOLAR	**2**.**37**	**1.08–5.21**	**0**.**030**
MIDPOSTERIOR BIPOLAR	**2**.**11**	**1.16–3.83**	**0**.**010**

## 8. Conclusions

The analysis of LV electrical potential in unipolar and bipolar configurations obtained by electromechanical mapping with the NOGA XP system (AEMM) in global, individual left ventricular wall and individual segment analysis has predictive value for a positive response to resynchronization therapy.

The mean bipolar potential and the sum of mean bipolar and unipolar potential are the predictive factors for a positive resynchronization response. The mean bipolar and the sum of unipolar potential obtained during the electromechanical mapping of the AEMM were the independent predictors of a positive response to resynchronization therapy.

In the analysis of the bipolar electrical potential of individual heart walls, the mean potential of the anterior and posterior walls was an independent predictor of favorable response to resynchronization therapy. In the unipolar signal analysis of individual walls, the mean interventricular septal potential was an independent predictor of favorable response to CRT.

In a detailed segmental cross-sectional analysis, independent predictive value for favorable response to the resynchronization therapy was shown by the bipolar potential of the basal part of the anterior wall and the medial part of the posterior wall.

## 9. Discussion

Although cardiac resynchronization therapy (CRT) is an established and effective treatment for systolic heart failure (3), it does not have the expected hemodynamic or clinical effects in approximately 30% of patients.

In recent years, in addition to standard resynchronization therapy, the frequency of using various techniques of pacing the conduction system has been increasing. Pacing of the His bundle has been reported in many studies to have similar electroradiographic and hemodynamic effects compared to traditional biventricular pacing (4, 5), although it is often associated with a slightly higher pacing threshold and LBBB correction is not possible in all cases (6). Alternatively, left bundle branch pacing (7), as well as combined pacing models including His bundle pacing and left ventricular pacing from the coronary sinus area are possible (8, 9).

Each of the methods mentioned above has its limitations and new pacing modalities have yet to be evaluated in randomized clinical trials. Therefore, the reasons for the lack of response to conventional resynchronization therapy are constantly being sought and efforts are being made define predictive parameters to identify those patients in whom we can expect a beneficial effect of biventricular pacing. One factor undoubtedly limiting the effectiveness of resynchronization is atrial fibrillation, which reduces cardiac output through loss of the hemodynamic effect of atrial contraction on the one hand, and a reduction in the percentage of biventricular pacing on the other hand. It is worth noting that seven patients (2.94%) in the study group had a history of atrial fibrillation. According to the inclusion criterion of sinus rhythm in the study group, atrial fibrillation in these patients was paroxysmal and did not constitute a clinical problem during the 6-month follow-up. Therefore, the study group did not consider advanced treatment strategies using node ablation, which are effective in the case of permanent form of this arrhythmia (10).

In our study, we focused on electrophysiological data obtained during electro-mechanical mapping (AEMM).

The potential recorded in the AEMM is a direct consequence of the electrophysiological properties of the myocardial tissue. It has not yet been used in predicting a response to resynchronization therapy. Electro-mechanical coupling underlies the heart's systolic function. The ability to depolarize and induce effective contraction is a property of all individual cardiomyocytes joined into structures that form a three-dimensional the myocardium model (11). The synchronized contractile activity of the cells determines the effectiveness of the global contraction of the heart. Cardiomyocytes die, and active contractile elements are lost and replaced by fibrous elements of connective tissue over the course of systolic heart failure. The changes involve the cardiomyocytes and the surrounding matrix (12). This occurs due to chronic or acute ischemia and secondary degenerative changes. Connective tissue in the form of scar tissue or foci of fibrosis cannot generate and transmit electrical potential or actively contract. These mechanisms underlie electrical remodeling leading to dyssynchrony and mechanical remodeling (13). The loss of contractile elements decreases the viability of specific areas of the heart; this translates into its geometry and, consequently, the load on the left ventricle, which reduces the pump's efficiency, leading to a decrease in the organ's systolic performance (14).

The myocardial viability assessment is widely used in diagnosing primary and secondary heart diseases and evaluating the adverse cardiotoxic effects of certain agents and exogenous substances, including drugs used in oncology and others. Viability assessments include methods using ultrasound, multi-row computed tomography, single photon emission tomography (SPECT), positron emission tomography (PET), and magnetic resonance imaging (MRI), and have been the subject of many studies (14–17). Vital parameter evaluation is used not only for diagnostic purposes but also to assess the effects of treatment—revascularization, stem cell use, or the effectiveness of systolic heart failure treatment. Imaging methods facilitate in analyzing heart's structure at the level of the entire organ, tissue structure, myocardial cell, as well as subcellular structures using nano-CT (12). In addition to structural analysis, functional disorders play an important role in the pathophysiology of heart failure. The presence of scar tissue or foci of fibrosis weakens contractile force, causes changes in shape and geometry, as well as preload and afterload, as described by Richardson et al. (18). Global scar mass determines the presence or absence of a beneficial effect of resynchronization therapy. According to Ojo et al. (19), damage of less than 15% of the left ventricular muscle and the absence of significant scarring in the posterolateral area are predictors of a favorable response to resynchronization therapy. Previously, similar findings were published by Ypenburg et al. (20, 21), also noting that the viability of 11 of 17 segments (65%) has predictive value in response to CRT.

In the analysis of our patient group, the predictive value of a favorable response to resynchronization therapy was demonstrated by the mean unipolar and bipolar potentials, as well as the sum of the mean unipolar and bipolar potentials of the individual, analyzed segments. In our study, we attempted to assess the predictive value of unipolar and bipolar electrical potentials demonstrated by mean unipolar and bipolar potentials, as well as the sum of mean unipolar and bipolar potentials of the individual, analyzed segments and their configurations in relation to a favourable response to resynchronization therapy. Considering the mechanics of left ventricular contraction under physiological conditions (22, 23), the analysis was performed at three levels: a general one reflecting the function of the myocadrium as a whole, the individual left ventricular walls: anterior, lateral, posterior and septal, as well as the most detailed one divided into individual segments in the basal and middle parts. At each level of analysis, the key baseline electrical potential determining the final effect of the implanted resynchronisation system was searched. For this purpose a predictive model was constructed for each of three levels of analysis. In the predictive model of the global analysis, the mean values of the bipolar potentials and the sum of the mean bipolar and unipolar potentials provided a predictive value for a favourable response to the resynchronisation therapy.

These parameters reflect global systolic potential and global left ventricular myocardial viability.

When analyzing the values of potentials for individual walls analyzed in the long axis of the heart, the value of the mean bipolar potential of the anterior and posterior walls demonstrated the predictive value of a favorable response to resynchronization therapy. In a detailed segmental cross-sectional analysis, the independent predictive value for favorable response to the resynchronization therapy was shown by the bipolar potential of the basal part of the anterior wall and the medial part of the posterior wall. Higher mean values and total potentials are associated with a smaller mass of scar tissue within the left ventricle, which is not able to generate electrical potentials. Scar tissue does not determine the effect of cardiac resynchronization therapy (CRT) solely by the size of its mass. According to the results of the target study (24), the location of the left ventricular lead within the scar is associated with a significant, fivefold higher mortality associated with the proarrhythmic effect previously reported by Nayaka et al. (25) and Sukla et al. (26). In addition, the scar alters the tissue's depolarization pattern through heterogeneous electrical conductivity of viable cell areas and fibrous tissue, which ultimately prolongs the activation time, as reported by Gardner et al. (27). According to Maffesanti et al. (28), a scar clearly affects the disruption of the relationship between electrical and mechanical activation. Scarring can also result in the loss of effective left ventricular pacing by increasing the pacing threshold. The mechanisms above underlie the risk of reduced resynchronization effect associated with the presence of scar tissue. Identifying the scar's size and location by available imaging methods, as well as the viability of myocardial tissue (15, 16, 17, 29) seems to be important per the cited facts. Electroanatomical mapping [AEEM], in addition to imaging methods, makes it possible to assess myocardial viability and perform scar localization analysis. The usefulness of electromechanical mapping was demonstrated in this regard by Maffesanti et al. (30), who related the mapping results to MRI as a reference method. Similar results were described by Pavo et al. (31), indicating the possibility of replacing MRI with electromechanical mapping in patients with contraindications to MRI. Sieniewicz et al. (14) also noted the possibility of identifying tissue that generates a bipolar potential in the 0.5–1.5 mV range during mapping, which is clearly lower than the potential values of healthy left ventricular tissue. This allows to differentiate healthy myocardium and regions of reduced vitality.

In previous studies, the values of unipolar and bipolar potentials characterized the healthy tissue, the scar area, and the transition zone differ in their values. According to Pavo et al. (31), the potential values characteristic of normal tissue in the unipolar system are above 15 mV while being greater than 1.9 mV in the bipolar one. For the infarct area, the unipolar signal is less than 5 mV, while it does not exceed 0.8 mV in the bipolar system. Intermediate values characterize the transition zone or incomplete wall infarction. By contrast, Van der Vleuten et al. (32) stress the lack of cutoff values in the data from the manufacturer of the NOGA XP system and report cutoff values for the unipolar infarct area potential of 11.0 mV. Data in this regard varies in the literature. Keck et al. (33) indicate a value of 4.5 mV, Botker et al. (34) 6.5 mV, and Koch et al. (35) 7.5 mV.

A meta-analysis of viability assessment based on the measurement of potentials recorded during electromechanical mapping against various reference methods was presented by Gyongyosi (36). Assessment of scar location in predicting response to cardiac resynchronization therapy is, of course, crucial, yet the identification of areas of reduced contractility showing clear, still preserved viability is no less important. The presence of these areas having some contractile reserve is a predictor of a favorable response to resynchronization therapy. Evaluation of this reserve is possible, on the one hand, by echocardiography after dobutamine administration, and nuclear medicine techniques (SPECT, PET and MRI) are also commonly used (37–39); on the other hand, the use of electromechanical mapping makes it possible to identify areas with electrical activity that are intermediate between healthy tissue and scar tissue (31). Van der Vleuten et al. (40) highlighted the usefulness of electromechanical mapping in identifying segments with reduced viability while noting the difficulty in defining unambiguous cutoff values for the potentials obtained.

In the study group, we found that a mean unipolar potential higher than 8.2 mV and a bipolar potential higher than 2.15 mV were predictors of a favorable response to resynchronization therapy. The electrical potential that produces a mechanical effect in the form of contraction and the perfusion of the analyzed area are two most important factors that guarantee the normal function of the myocardium. Identifying these mechanisms, their disorders, as well as their reserves seems crucial for identifying potential responders to resynchronization therapy. Nevertheless, it seems that further research using various methods to study these phenomena, including the evaluation of generated electrical potentials in the key areas of the myocardium, will help increase the percentage of patients benefiting from resynchronization therapy.

## 10. Study strengths and limitations

The value of this study was the use of the highly accurate NOGA XP electromechanical mapping system, innovative in the evaluation of CRT response. It should be noted that electromechanical mapping was performed exclusively for this project. The main limitation of the study is the relatively small group of patients studied. However, due to the invasive nature of the mapping, recruitment to the study was halted when statistically significant correlations occurred.

Currently, new pharmacological strategies are being used in the treatment of chronic heart failure, i.e., phlazines (41) and sacubitril/valsartan (42, 43). It should be noted that the patients included in our study were not using sacubitril, which received the US Food and Drug Agency (FDA) approval in 2015 and in Poland in 2016, while recruitment to the study was already underway. Also, the patients included in the study were not using flozins. Based on the results of the DAPA-HF trial, the European Medicines Agency (EMA) and US Food and Drug Agency (FDA) approved dapagliflozin in 2020 for the treatment of patients with symptomatic chronic HFrEF (41).

## Data Availability

The raw data supporting the conclusions of this article will be made available by the authors, without undue reservation.
